# Context-Dependent Memory of Motor Sequences

**DOI:** 10.5334/joc.152

**Published:** 2021-02-17

**Authors:** Markus Schmidt, Christian Frings, Tobias Tempel

**Affiliations:** 1 Ludwigsburg University of Education, DE; 2 University of Trier, DE

**Keywords:** context change, intentional context stimuli, incidental context stimuli, context-dependent learning, context-dependent retrieval

## Abstract

To examine influences of context changes between encoding and retrieval of motor sequences, we varied a number of encoding and retrieval features in a two lists approach. Participants consecutively learned two sets of three-finger movements at two different computer working places, all enacted with fingers of the right hand. We varied keyboard and display orientation, stimuli, background color, response keys, position of the hand, and the used PC between the two sets. A final free recall test comprised either the same context features as present during study of the first item set or the ones present during study of the second item set or novel test context features. Results showed significant differences in overall recall performance between test conditions, indicating that context features of study episodes guided retrieval of motor sequences. In addition, the number of recalled items varied as a function of output position. Test context elements comprising context features of the first item set study episode were associated with initially lower but subsequently nearby stable recall performance, whereas test features comprising context elements of the second item set study episode were associated with initially higher and subsequently decreasing recall performance. This implies that a context reinstatement for list-1 items during the test phase does not immediately enhance accessibility of those items. However, access is subsequently facilitated over the course of retrieval attempts.

The context-dependency of memories typically is a subtle phenomenon. Imagine the everyday situation where you stand in the cashiers waiting line of your local supermarket. You can’t remember where you parked your car in the supermarket parking lot while trying to think of it inside. But standing on this parking lot after leaving the market you immediately recall the position of your car. This (environmental) context-dependent memory phenomenon received empirical evidence in Godden and Baddeley’s (1975) classical study, wherein they created two remarkably different natural environmental situations – on land and under water. Memory for word lists learned on land was best if the lists were recalled on land, word lists learned under water were best recalled under water. Lists learned on land getting recalled under water or lists learned under water getting recalled on land produced reliably worse recall. So, memory performance (for word lists in that case) depended on the contextual match between the retrieval and the encoding situation, i.e. recall was better if the environment of original learning had been reinstated.

Context has a fundamental impact on memory, effects of environmental context changes on subsequent recall were found for room changes (Smith, Glenberg, & Bjork, 1978), for background music (Smith, 1985), for odors (Pointer & Bond, 1998), and the background color of a monitor (Isarida & Isarida, 2007), to name just a few examples. Context change effects also expand to posture (Rand & Wapner, 1967). A pose as a contextual (background) cue could reflect a temporal-spatial or a social-emotional context as well as an internal state driven element just present in your mind. So beyond external (physical) attributes, there may be an internal or mental contextual cue. Bower (1981) for example examined mood-state-dependent memory in recall of word lists. Sahakyan & Smith (2014) referred to mental context as: “our constantly changing blend of thoughts that evolves in response to encoding and retrieval of other events” (p. 86). We here always will refer to environmental (external) context, whenever we speak of context, or name explicitly which context we speak of.

Evidence for a context-dependency of motor memory is much more limited. Ruitenberg, De Kleine, Van der Lubbe, Verwey, & Abrahamse (2012) examined context-dependent learning in the discrete sequence production task. In this task, participants typically face two sequences of two to seven stimuli in a ﬁxed order and respond by means of spatially compatible key presses. Ruitenberg et al. (2012) presented two differently colored stimuli simultaneously, one relevant and one irrelevant. Performance was impaired when the irrelevant information was changed in the test phase, but not when it was removed, showing that sequence learning in the discrete sequence production task is context-dependent and that the context dependency was modulated by the amount of practice; the context effects diminished as practice increased.

Wright and Shea (1991) used a different approach to examine contextual dependencies during motor skill acquisition. They proposed a distinction for the environmental dimension into intentional and incidental stimuli. Whereas intentional stimuli “were defined as essential for achieving skilled performance”, incidental stimuli “were defined as those that have the potential to become associated with specific tasks due to their selective presence in the learning environment” (p. 361). In their Experiment 1, they manipulated the proportion of intentional and incidental stimuli between learning and test. The same intentional and same incidental stimuli (as in the learning phase) were given at test in one condition (context reinstatement), the same intentional but switched incidental stimuli in another condition, and finally no intentional but same incidental stimuli in a third condition. Participants were told to make use of the intentional stimuli for planning and enactment of the keypress sequences, but the incidental stimuli were not mentioned at all. Their performances showed less errors in the reinstatement condition than in the same intentional but switched incidental stimuli condition. Both conditions involving a change of stimuli at test showed reduced performance as compared to context reinstatement. Memory for motor skill performances showed a reliable context-dependency.

Tempel and Frings (2016) examined directed forgetting (the intentional effort to forget previously encoded information) in motor memory and found evidence for a context-dependency of motor memory. Two lists of sequential finger movements served as item material. Each sequential finger movement consisted of consecutive key presses from four fingers of the right hand. A cost effect of directed forgetting (i.e. the *forget*-cued list is recalled worse than the *remember*-cued list) for List one (L1) only emerged in Experiment 2, which involved a three-minute break between L1 and List two (L2) along with different response keys for L2 as for L1. Test phase response keys were those of L2. The motor sequences recall in this study might have depended on the changes in external (context) features, such as different response keys and a three-minute break between L1 and L2, that also may have facilitated a change in the internal context as a consequence of the forget instruction for L1.

## The present study

Previous studies on the context dependency of motor memory focused on incidental motor skill acquisition. Wright and Shea’s (1991) distinction between intentional and incidental stimuli only referred to directing attention to features that guided performance of a motor task (intentional) versus background features (incidental). However, learning motor sequences is not always a mere by-product of repeatedly performing a certain task. In many situations, there is an intention to learn and retain certain motor sequences, for example, when practicing how to play a piece of music on an instrument. Here, we examined effects of switching intentional and incidental stimuli, as defined by Wright and Shea (1991), on an intentional learning setting of motor sequences.

With respect to the results of Tempel and Frings’ (2016) directed forgetting study, that might reflect evidence for a context-dependency of motor memory, we used a similar design in this study, this time focusing only on contextual changes between two lists – and at test. In the present study, participants learned two lists of sequential finger movements consecutively, both followed by a ten-minute break. We designed two different sets of eight sequences, with each sequence consisting of three consecutive keypresses of the index, middle, ring finger or pinkie of the right hand. In all of our four experimental conditions, these two sets – L1 and L2 – were learned in two notably different encoding situations, both including a variety of remarkably different intentional and incidental stimuli. This contrasting environmental manipulation between L1 and L2 was meant to induce attention on these intentional and incidental contextual stimuli.

**Figure 1:**
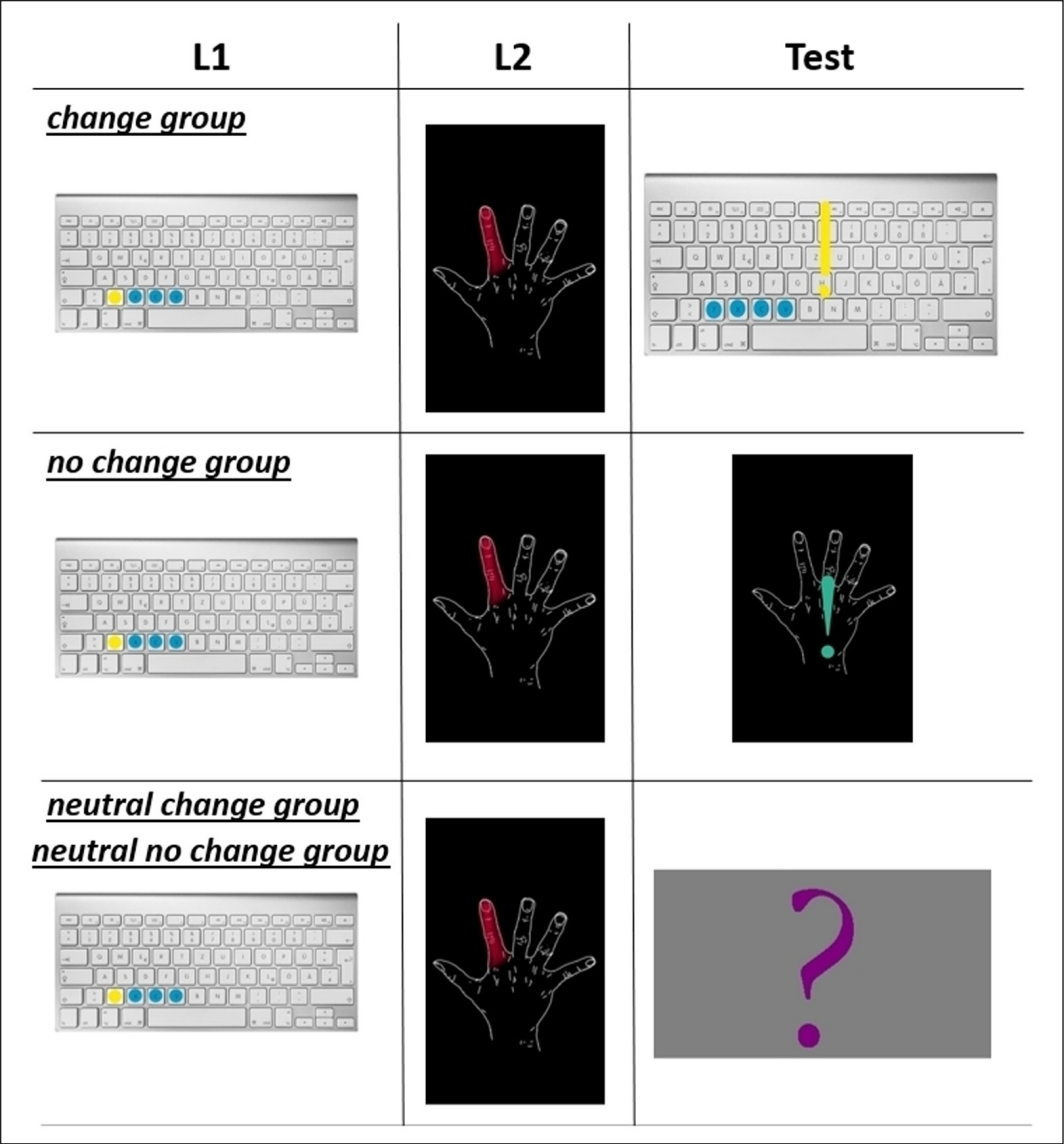
Diagram of the visual intentional cues in our four experimental groups at L1, L2 and at test, groups and experimental phase labeled and separated in boxes. Pictures show an example of the groups corresponding displayed intentional stimuli at encoding and test, including their display orientation (vertically vs horizontally). For the two *neutral* groups a question mark highlighted each input task at test, for the other two groups an exclamation mark demanded for the sequences input. Colors at test in the *change* and *no change* groups were held in the corresponding group test context, colors for the *neutral* groups were new ones as compared to L1 and L2 (see the *Material* section). The *neutral* groups differed with regard to used PC, display orientation, response keys and keyboard position from each other, which were the same as study of L1 (*change group*) or L2 (*no change group*).

We then created four different test conditions. The *change group*, constituting the first test condition, switched the intentional and incidental test context completely back to the L1 encoding situation, including the L1 response keys then being the test response keys. This group, thus, re-established access to the representation of the L1 encoding context. The *no change group* as the second test condition remained at test identical to the L2 encoding situation, including the L2 response keys then being the test response keys. So, there was no intentional or incidental contextual change in this group from L2 encoding to the test situation. The remaining two test groups, *neutral change group* and *neutral no change group* were designed similarly. The *neutral change group* also switched the incidental test context from L2 encoding back to the L1 encoding context, but not the intentional test context. The complete displayed test cues and instructions – fonts and used colors – were held neutral, as compared to L1 and L2. The same neutral colors and fonts were used at test in the *neutral no change group*, wherein the incidental test context remained at the L2 context, despite these changes in the visual stimuli. In both *neutral* groups, the visual stimuli thus changed to novel intentional cues (fonts, colors, test cues), that is, these stimuli did not match either L1 or L2. Beside this mismatch in the intentional test stimuli set, the *neutral change group* additionally changed the used PC, display orientation, response keys and keyboard position back to the ones used for L1 learning. For the *neutral no change group* these incidental stimuli remained the same as for L2 learning (see ***Figure 1***).

With regard to intentional learning, recently, the theory of *the two faces of memory retrieval* (e.g., Bäuml, 2019; Bäuml & Samenieh, 2012a, 2012b) posited that context changes between encoding and test modulate how recall performance develops over recall trials. Recall performance either improves or worsens over test trials because the first trials of a test either facilitate subsequent retrieval of further items (via re-establishing access to a representation of the common encoding context) or inhibit further items (when the context did not change). Bäuml and Samenieh (2012a, 2012b) proposed a two-factor account, explaining why retrieval can be a self-limiting process in the absence of a contextual change, but be self-propagating in its presence. According to their account, the original encoding context is deactivated after a change in external or internal context. Initial retrieval of some of the original context items then reactivates this context, and as a consequence, retrieval of the other context-related items becomes easier over the course of retrieval attempts, self-propagating. In the absence of a context change, the original encoding context remains active and initial retrieval of some related items can inhibit or block access to the items of the other context. Retrieval becomes a self-limiting process. For the present investigation, therefore, we also analyzed how recall performance developed over test trials by comparing the first with the second half of the test trial responses.

According to Bäuml & Samenieh’s (2012a, 2012b) *two faces* account, we expected recall for the *no change group* as being a self-limiting process. For the absence of a contextual change, the L2 encoding context should remain active and initial retrieval of some items related to this context should inhibit or block access to further items. Initial recall should be high but decline subsequently. In contrast, recall in the *change group*, wherein context changed after L2, should be self-propagating. Initial retrieval of some of the L1 context items should reactivate this context, and subsequent retrieval of the other L1 context related items should become easier. So, according to the *two faces* account (e.g., Bäuml, 2019; Bäuml & Samenieh, 2012a, 2012b), we expected initial recall in the *change group* to be lower, but subsequently increasing or at least stable, not declining, as we expected it to be in the *no change group*.

We expected recall in the *neutral no change group* as also being a self-limiting process. Recall for this group, wherein we switched the visual stimuli to neutral, should behave similarly to the *no change group*. The maintained environmental L2 context features at test should not get into a cue conflict with a displayed neutral stimuli set. Neutral visual cues also should not suffice to induce a context change. The test, furthermore, used the same response keys as L2. So, the L2 encoding context should remain active. Initial retrieval of some items related to this context should inhibit or block access to further items. Initial recall should be high but decline subsequently.

With the *neutral change group*, we explored whether the L2 context would even remain active with a further switch in incidental stimuli. Thus both, the environmental feature set and the response keys, were switched back to L1. The (intentional and incidental) displayed stimuli were the same as in the *neutral no change group*. So, this group constitutes a not completely reinstated condition. These neutral display stimuli here might prevent a contextual change back to L1, that is, switched environmental features and the response keys from L1 might not suffice for a contextual change back to L1. So, the L2 encoding context as the last context should remain active. Initial retrieval of some items related to this context then again would inhibit or block access to further items.

## Method

### Participants

One-hundred-and-thirty-six students (mean age = 22.9) at the PH Ludwigsburg, University of Education, participated in the experiment. Each of the four experimental groups, thus, contained thirty-four students. An a priori calculation of the required sample size with 1-β = .9, α = .05 and an estimated effect size based on prior motor memory results (e.g. Tempel & Frings, 2016, 2017, 2019) of f = .2 resulted in one-hundred-twenty-eight participants. We conducted the experiment in groups by three participants and also ran some more participants to account for drop outs. All students were paid ten Euros each for their participation.

### Design

We manipulated the test context by comparing four groups that differed with regard to matching or, respectively, non-matching intentional and incidental stimuli. In the *no change* group, intentional stimuli (i.e. visual stimuli representing the to-be-performed sequences) and incidental stimuli (i.e. used PC, display orientation, instructions fonts and colors, response keys, and keyboard position) remained the same as during learning of L2, whereas intentional as well as incidental stimuli switched back to those present during learning of L1 in the *change* group. In a third and fourth group, no intentional stimuli from either L1 or L2 were repeated but, instead, neutral visual stimuli prompted execution of the learned motor sequences in these *neutral* groups. Incidental stimuli remained the same as during learning of L2 in the *neutral no change* group but switched back to those present during learning of L1 in the *neutral change* group.

### Material

The experiment was conducted using different PCs with standard German QWERTZ keyboard layouts. The software PsychoPy in version 1.90.1 (Peirce et al., 2019) served for running the experiment. The items consisted of two lists of eight three-finger movements of the index-, middle-, ring finger or pinkie of the right hand (see **Appendix A**). The three-finger movements were to be enacted on the second lower row of the horizontally orientated keyboard (keys were Y, X, C and V) for L1. L2 keyboard position was flipped ninety degrees, so the sequential finger movements had to be enacted on the second lower row of the number pad, keys were Num_Decimal, Num_3, Num_6 and Num_9.

**Figure 2:**
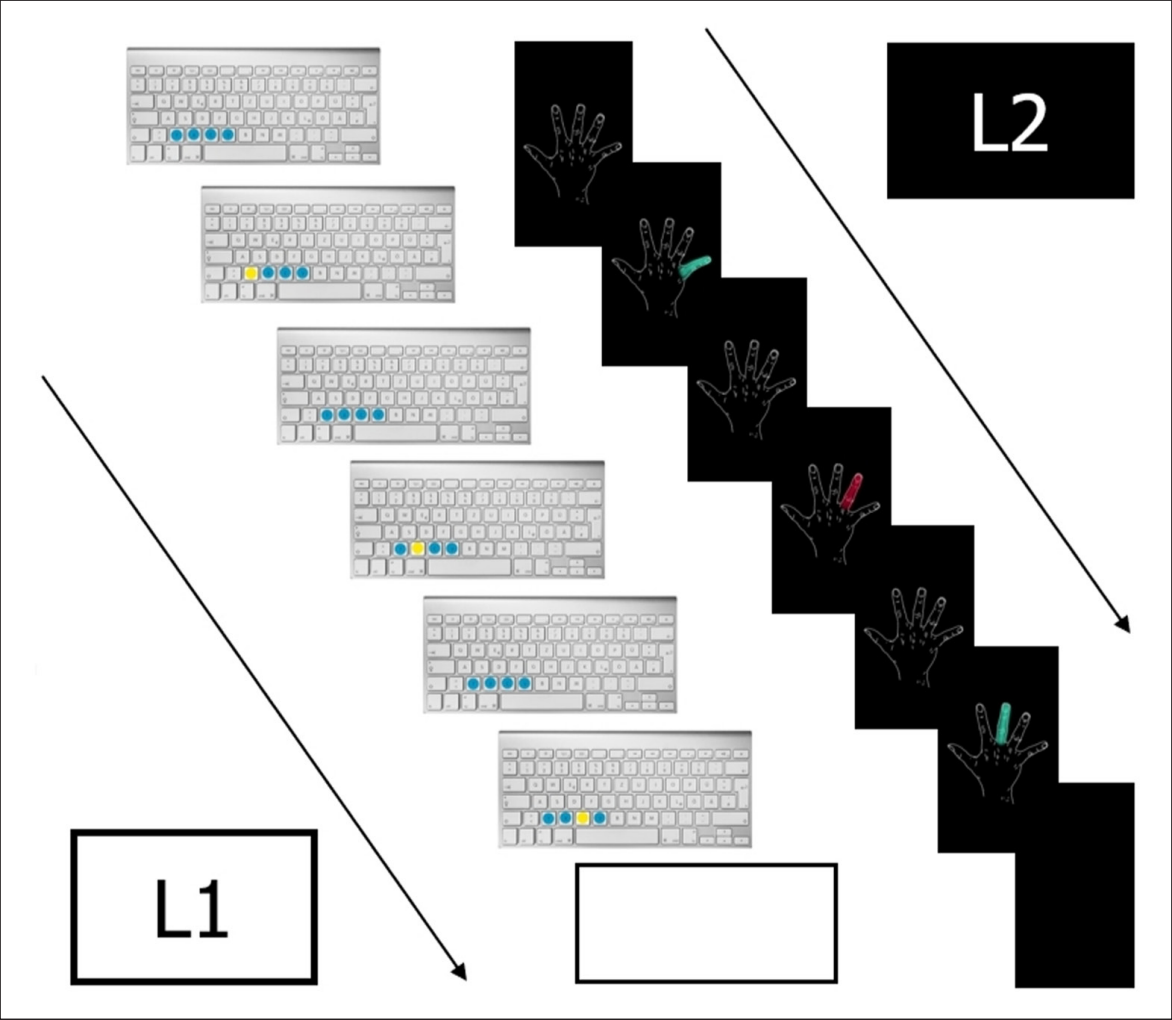
The Figure depicts one displayed symbolic item trial for both, L1 (left side) and L2 (right side). L1 intentional stimuli were the “moving” keys on a grey symbolic Mac-Keyboard. L2 intentional stimuli were the “moving” fingers in a drafted symbolic hand. Both items were displayed in a colored animation followed by a blank screen pause. Participants had to enter this sequence then, before the next sequence gets animated. At the beginning of the animation, a picture of the keyboard including the four not moving reaction keys marked in blue or a drawing of the corresponding hand appeared for 1500 milliseconds (ms). Then, an animation of the keys or the fingers in the hand showed three consecutively flashing keys or fingers. The index finger in L2 was highlighted in dark red, middle finger was colored in a light green, the ring finger was colored dark red again, pinkie again in light green; 200 ms per colored finger flash followed by 200ms for the uncolored hand drawing before the next moving finger. All moving keys in L1 were highlighted in yellow, the three not moving keys in between were given in blue; also 200 ms per color flash followed by 200ms for the four blue (not moving) keys before the next colored key. Once the animation disappeared, participants could perform it immediately by sequentially pressing the three corresponding keys.

To create two contextually distinct encoding conditions, we varied several physical features. During the learning phase, the intentional stimuli, i.e. the animation of the finger sequences, were in color highlighted keyboard buttons for L1 and in color highlighted fingers in a drafted symbolic hand for L2, indicating the order of the sequence enactment in color highlighted animation (see ***Figure 2***).

We used two nearest to the complementary different colors for each list. Additionally, the used colors for L1 were different to the ones for L2. L1 move indicating color was yellow, not moving keys were blue. Thus, the four reaction keys Y, X, C, and V were highlighted on the display throughout the whole learning trial. L2 move indicating color was red and green for fingers side by side. The visual intentional stimuli, thus, were the “moving” keys on a grey symbolic Mac-Keyboard in L1 or the “moving” fingers in a drafted symbolic hand in L2, both in a displayed color animation followed by a blank screen pause, before the next sequence gets animated. At the beginning of the animation, a picture of the keyboard including the four reaction keys marked in blue or a drawing of the corresponding hand appeared for 1500 milliseconds (ms). Then, an animation of the keys or the fingers in the hand showed three consecutively flashing keys or fingers. The index finger in L2 was highlighted in dark red, middle finger was colored in a light green, the ring finger was colored dark red again, pinkie again in light green; 200 ms flashing the color highlighted finger followed by 200ms for the uncolored hand drawing before the next “moving” finger. All move indicating keys in L1 were highlighted in yellow, the other three response keys in between were given in blue. Each yellow highlighted key plus the three not moving (blue) keys was flashing 200 ms, followed by 200ms for the four blue (i.e. not moving) response keys before the next yellow highlighted key. Once the animation disappeared, participants could perform it immediately by sequentially pressing the three corresponding keys. Feedback about the performed sequence was given for 800 ms, indicating wrong finger movements by displaying: “Fehler!” (English: “Error!”) in the center of the screen. Error feedback was given to foster encoding accuracy, following previous studies with the same kind of learning material (e.g. Tempel & Frings, 2016, 2017, 2019). Correctly entered sequences were followed by 800 ms blank screen instead. After further 800 ms blank screen, the next trial started (see ***Figure 2***).

**Figure 3:**
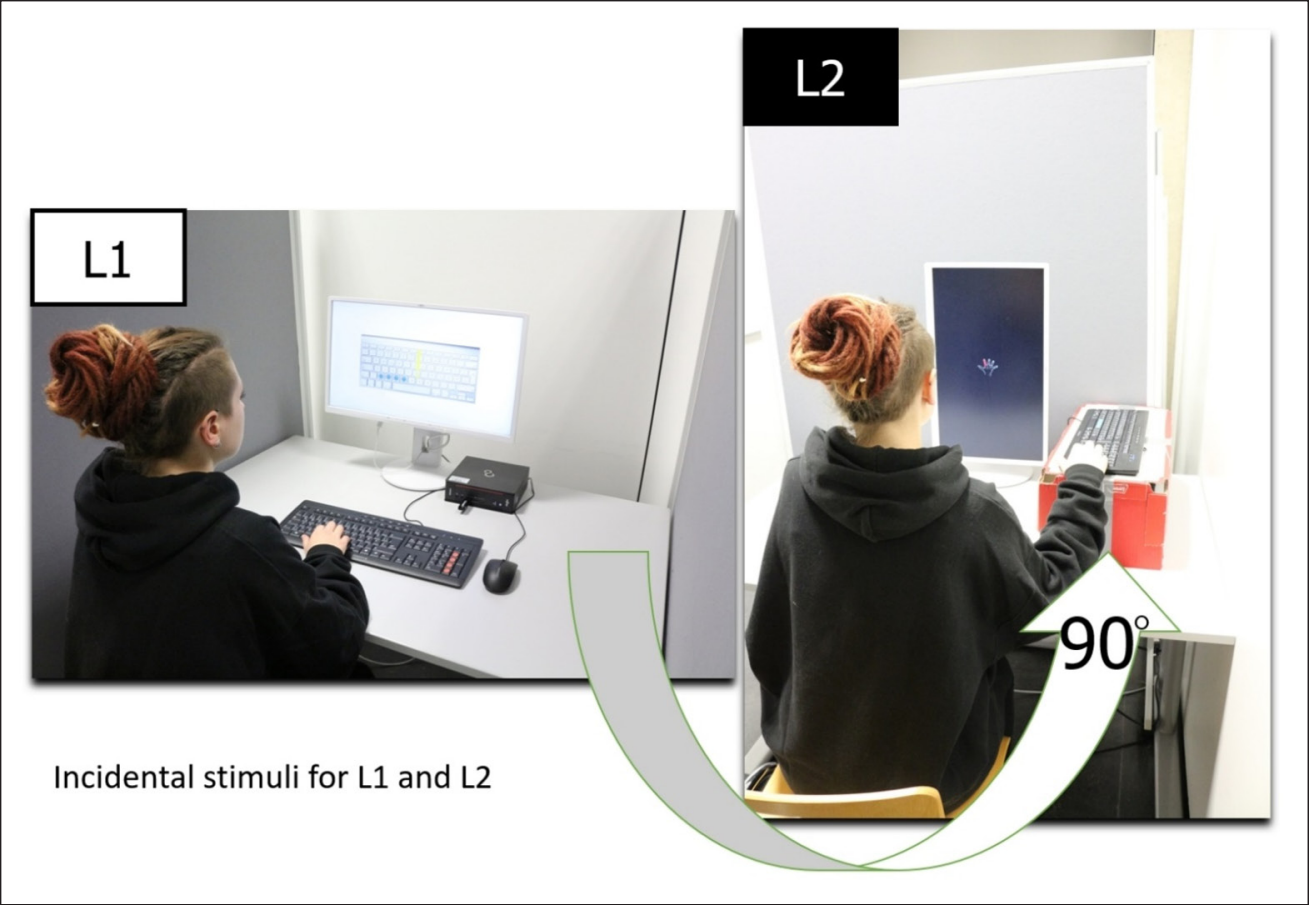
Photographies illustrating the different L1 and L2 incidental and intentional stimuli. L1 picture shows a *change group* participants’ test trial. L2 picture shows a learning trial.

We also varied several incidental features between lists. L1 background color was white, instructions were given in a black font, the symbolic keyboard picture was given in a grey Mac-Laptop-Layout. L2 background color was black, instructions and drafted symbolic hand were given in a white font. L2 display orientation was vertical, L1 display was in a horizontal position. Keyboard orientation for L1 was horizontal, L2 keyboard orientation vertical. Moreover, in L2 the keyboard itself was placed on a small shelf of that height, that participants could reach the response keys only in a slight downward position of their hand, in which they were told to leave their elbow on the table during the whole learning procedure and their fingers just over the keys. On a (standard) horizontal positioned keyboard (L1) the hand has a slight upward position. The experimenter explained and supervised this uncommon hand position. Participants then were instructed not to focus on their hand in the following learning phase but on the displayed animation instead and to keep the hand in this position. Finally, the whole separated working place for L2 had a ninety-degree shift compared to L1 (see ***Figure 3***).

### Procedure

The experiment consisted of three phases: L1 learning, L2 learning and a final memory test. First, general instructions were given on the computer screen and face-to-face verified by the experimenter. Participants then had a practice trial of two out of the lists items, to become familiar with the sequence animation and the corresponding response keys. Akin to Wright & Shea (1991), participants were instructed to use the intentional stimuli for the encoding of the sequential finger movements, but the incidental stimuli were not mentioned at all. Afterwards L1 learning began. All eight L1 items were learned eight times in succession, in a random order. Subsequently a ten-minute break began, wherein participants were demanded to leave the room. L2 learning then took place in a different separated working place in the same room on another PC that had a spatially ninety-degree rotation, compared to the L1 working place. All eight L2 items also were learned eight times in succession in a random order, then the second ten-minute break took place. Participants again were instructed to leave the room after L2 learning for ten minutes, as they did after L1 learning. So, both learning trials had the same procedure. Then participants were tested with either the same or different incidental and/or intentional contextual cues compared to L2 learning, depending on their experimental group assignment.

In this final test phase, we had a *change group* condition, wherein we induced a(n) (external) context change, by switching the test context back to the L1 intentional and incidental stimuli feature context, or we maintained the L2 intentional and incidental stimuli feature context, constituting thereby a *no* (context) *change group* condition. Two further conditions were designed along this *change* or *no change* pattern, that also differed in their reinstated or maintained features. The third condition, *neutral change group*, also switched the test context features back to the L1 incidental context but with neutral visual stimuli. The complete displayed test instruction and test cue set here was held in a neutral color and font as compared to the ones used in L1 and L2, but the test used the same PC, display orientation, response keys and keyboard position as L1. The fourth condition, *neutral no change group*, maintained the L2 incidental context features at test but also had neutral visual stimuli – the same ones as the *neutral change group*. So, the complete displayed test instruction and test cue set here also was held in a neutral color compared to the ones used in L1 and L2, but the test used the same PC, display orientation, response keys and keyboard position as L2.

Recall for all sixteen items (both lists) then was assessed in a self-paced free recall test. Recall was instructed explicitly in any order the items come to mind, no matter what list the items belonged to, but with the intention to recall all items of both lists. Each sequence input (three keys pressed in succession) was prompted by the corresponding test cue (a question mark or exclamation mark in front of visual stimuli matching L1, visual stimuli matching L2 or a plain grey). Test trials were separated by 500 ms blank screen. The free recall test was not limited in time, but participants had to enter sixteen sequences (consisting of three consecutive keypresses each).

## Results

To inspect the overall recall performance under the perspective of the *two faces* account (Bäuml, 2019; Bäuml & Samenieh, 2012a, 2012b), we divided the sixteen serial recall positions of our four experimental conditions into two groups. The serial recall of the first eight recalled items are labeled T1 in the following. Accordingly, recalled items of the serial positions nine to sixteen, the second eight recalled items, are labeled T2 in the following.

We then conducted a 4 (*no change, neutral no change, neutral change, change*) *groups* × 2 (T1, T2) *recall* ANOVA with repeated measures on the last factor. The main effect of *groups* was not significant, with *F*(3, 132) = 2.34, *p* = .076, η_p_^2^ = .051. The main effect comparing T1 and T2 *recall* was significant, with *F*(1, 132) = 43.25, *p* < .001, η_p_^2^= .247. The interaction *groups* × *recall* was significant, *F*(3, 132) = 2.84, *p* = .041, η_p_^2^= .061, caused by the *change group*, which showed a different result pattern compared to the other three group conditions. All T1 and T2 recall differences for these three groups were significant, but the *change group* showed no such reliable difference (see ***Table 1***).

**Table 1:** T-tests for T1 and T2 recall differences for our four experimental groups.


CONDITION GROUP	*t*	df	*p*	SIGNIFICANCE

*no change*	5.1	33	<.001	**

*neutral no change*	3.2	33	.003	**

*neutral change*	4.5	33	<.001	**

*change*	.94	33	.36	n.s.


**Figure 4:**
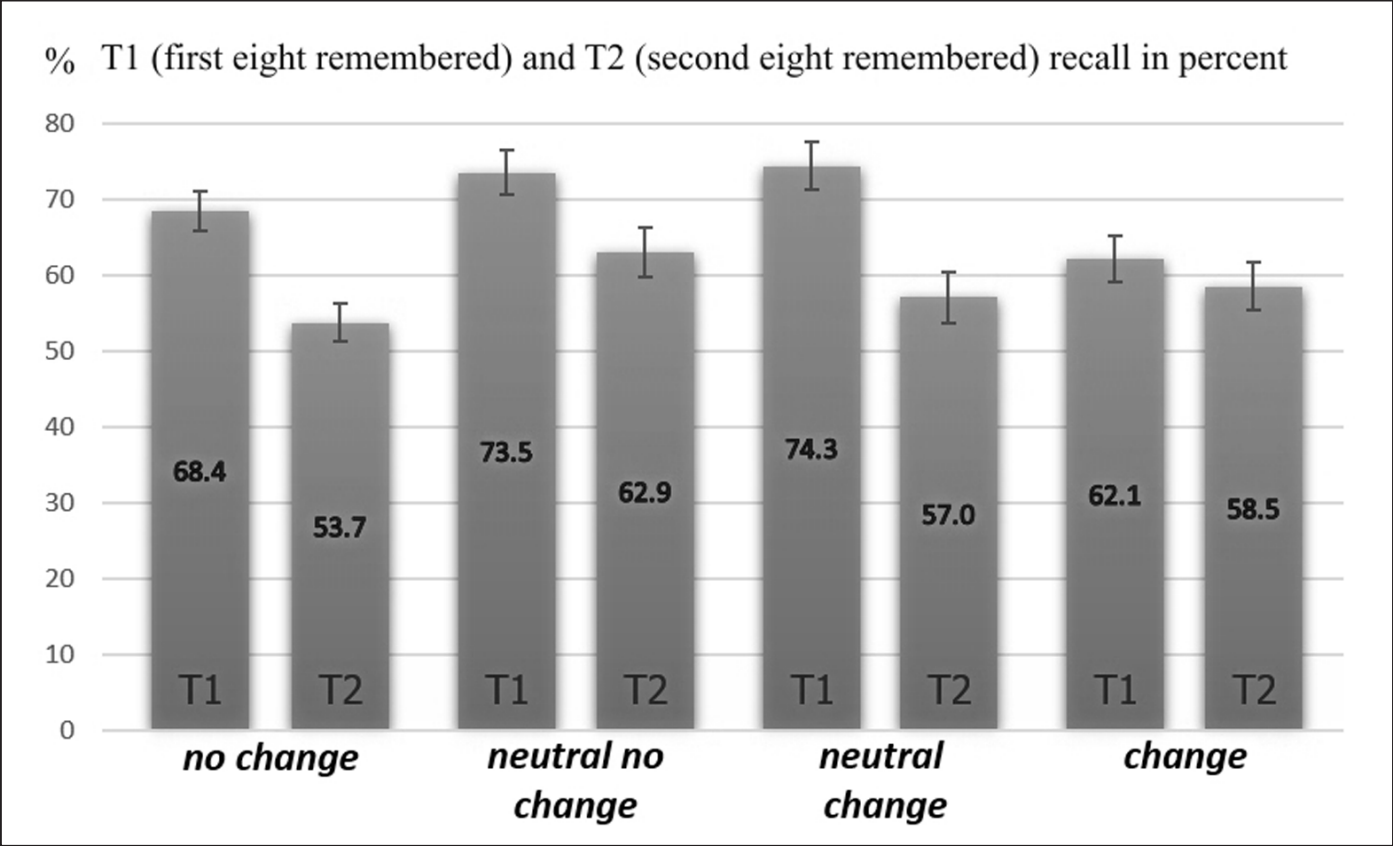
T1 and T2 recall rates of our four experimental condition groups in percent. Error bars represent ±1 S.E.M.

So, the recall pattern of the three experimental groups *no change, neutral no change*, and *neutral change* was similar, but these three groups differed remarkably from the recall pattern of the *change group*^1^ (see ***Figure 4***).

## General Discussion

The expected recall patterns for our two main conditions, the *no change* and *change group*, emerged. According to the *two faces* account of (context-dependent) retrieval (e.g. Bäuml, 2019; Bäuml & Samenieh, 2012a, 2012b), we expected recall for the *no change group* to be a self-limiting process. For the absence of a contextual change at test, the L2 context should remain active and initial retrieval of some items related to this context should inhibit or block access to other items. Initial recall should be high but decline subsequently. This pattern was observed.

Recall for the *change group*, in which intentional and incidental stimuli changed after L2 completely back to the L1 context, should be self-propagating for the presence of a (true) context change at test. Initial retrieval of some items then should facilitate subsequent retrieval. We expected initial recall in the *change group* to be lower, but subsequently staying at least nearby stable, not declining as in the *no change group*. This pattern was observed.

The *neutral no change group* and *neutral change group* showed the same recall pattern as the *no change group*. This suggests that the at test maintained L2 or reinstated L1 environmental context features in these groups did not to get into a cue conflict with a neutral (intentional and incidental) display stimuli set. Neutral display stimuli did not suffice to induce a context change. The test in the *neutral no change group*, furthermore, used the same response keys as L2. So, the L2 encoding context should remain active and initial retrieval of some items related to this context should inhibit or block access to further items. Initial recall should be high but decline subsequently. This pattern was observed.

A comparison of the recall pattern in the *neutral change group* with the *change group* showed the importance of the visual intentional (recall cues) and incidental stimuli (instructions font and colors, background color) set for the occurrence of a context effect. Only a complete change of intentional as well as incidental stimuli after L2, back to all features present during L1 learning, eliminated the typical pattern of a strong decreasing recall from T1 to T2, which suggests that only this combined mismatch was a true context change. This underlines the importance of the intentional as well as the incidental (visual) stimuli for the occurrence of a context-dependent memory effect and further shows the influence of intentional and incidental context features given at encoding and test for an intentional learning and recall setting of motor sequences. Moreover, the proposals of the *two faces* account of (context-dependent) retrieval (Bäuml, 2019; Bäuml & Samenieh, 2012a, 2012b) critically assume, that the degree of context change determines the relative contributions of the mechanisms blocking/inhibition and contextual retrieval. That means, if the degree of context change is low or moderate only, the beneficial effect from T1 on T2 recall may be low or even absent. We reported a detrimental effect from T1 on T2 for our *neutral no change group* and the *neutral change group*, which also could be labeled *small context change* conditions. For our *change group* we reported a neutral effect. This pattern fits with the *two faces* account, implying that the size of the induced contextual effects for our *change group* appears to be moderate only, so that there is reason to expect a neutral rather than a beneficial effect for this group.

Ruitenberg et al. (2012) in their incidental learning setting suggested, that the effects of context in the discrete sequence production task not only reflect a facilitation of retrieval due to reinstatement, but also the learning of an effective dealing with irrelevant stimuli, what they refer to as context-dependent filtering. The switched intentional and incidental stimuli in our intentional motor skill acquisition study were used to accentuate a contextual shift. So, there was no way of filtering them out, for they defined the very context itself. If they are to be labeled, then they are rather distracting than irrelevant. Laub & Frings (2018) found a distractor-based action control effect that was modulated by encoding specificity. The distractor-based retrieval process in their experiment depends on the contextual similarity between the encoding and the retrieval context. Laub & Frings (2018) found clear evidence for the encoding specificity of this process. Distractor-based retrieval was found only in conditions with the same number of distractors in the prime and in the probe, if the encoding and retrieval context were contextually similar. Solely for conditions with the same number of distractors in the prime and the probe the typical pattern indicating distractor-based retrieval occurred. If the number of distractors changed between the encoding and retrieval context, no distractor-based retrieval occurred. This is similar to our observed results. Only for the condition with the identical “distracting” intentional and incidental stimuli at encoding and test – our *change group* – a true context change emerged and the expected recall pattern according to theory of *the two faces of memory retrieval* (e.g. Bäuml, 2019; Bäuml & Samenieh, 2012a, 2012b) occurred.

Wright & Shea (1991) showed in their Experiment 2, that changes in the intentional and incidental stimuli given at test within a reduced task difficulty setting (by switching from four- to three-key sequences) did not disrupt motor performance any more. Participants were able to perform the three-key sequences even in the absence of any intentional stimuli (third condition). This indicates developed (strong) associations between the incidental stimuli and the sequences that could be used at retrieval (in case of a cue conflict), even though participants just were instructed to attend to the intentional stimuli as an acquisitional aid. Our results also suggest developed strong associations between our incidental stimuli and the sequences, leading to a perception of the respective lists sequences as two different contexts that could get reinstated at test.

Further studies on these observed contextual recall dependencies of motor sequences could clarify the role of the hand position and, more generally, which incidental and intentional features are necessary at all to result in contextual effects? Future work may also like to elucidate the role of contextual effects for retrieval dynamics of motor sequences when context is changed more drastically. Under such conditions of a drastic context change, even a beneficial effect from T1 to T2 should arise, at least if the theory of *the two faces of memory retrieval* applies to the present situation and T1 retrieval attempts last long enough to facilitate contextual accessibility, i.e. the lists are long enough.

Taken together, further research is needed to clarify the role of intentional and incidental context features as contributors for a perceived environmental context change. The present results suggest that only a combined mismatch was a true context change.

Our results have implications on the practical side of intentional motor skill acquisition. Imagine, for example, a school orchestra and their music teacher learning pieces of music for the annual school concert. Learning all the concert pieces of music in one and the same environmental context, which is also rather similar than different to the test context (i.e. the concert hall), will result in a high accessibility of the trained motor skills at that annual school concert. Performance there will show less errors, if the “test” environment will not be perceived as an environmental context change as compared to the learning environment.

## Data Availability

*https://osf.io/knqag/?view_only=0847a7bbd3964d62bf504ec6e1cd04fe*.

## Footnotes

In additional analyses, recall positions were divided into four blocks, each comprising four consecutive recall positions. When looking at the three groups *no change, neutral change*, and *neutral no change*, there was a strong linear trend indicating a decrease of recall success, *F*(1, 99) = 96.01, *p* < .001, η_p_^2^ = .493, which did not differ between groups *F*(2, 99) = 1.184, *p* = .310. Although there also was a linear trend in the *change group, F*(1, 99) = 7.99, *p* = .008, η_p_^2^= .195, it was significantly weaker in contrast to the other three groups, *F*(1, 134) = 5.60, *p* = .019, η_p_^2^ = .04.

## Appendix A

Items.

**Table:** L1 – items.

ITEM	FIRST FINGER	SECOND FINGER	THIRD FINGER

1	index finger	middle finger	pinkie

2	index finger	pinkie	ring finger

3	middle finger	ring finger	pinkie

4	middle finger	ring finger	index finger

5	ring finger	index finger	pinkie

6	ring finger	index finger	middle finger

7	pinkie	index finger	middle finger

8	pinkie	ring finger	index finger

**Table:** L2 – items.

ITEM	FIRST FINGER	SECOND FINGER	THIRD FINGER

1	index finger	ring finger	middle finger

2	index finger	ring finger	pinkie

3	middle finger	index finger	ring finger

4	middle finger	index finger	pinkie

5	ring finger	pinkie	index finger

6	ring finger	pinkie	middle finger

7	pinkie	middle finger	index finger

8	pinkie	ring finger	middle finger
